# Musical expectations modulate declarative memory formation

**DOI:** 10.3758/s13423-026-03020-4

**Published:** 2026-09-23

**Authors:** Naama Benes, Daniel Y. Panitz, Shai Gabay, Avi Mendelsohn

**Affiliations:** 1 Sagol Department of Neurobiology, University of Haifa, Haifa, Israel; 2 The Institute of Information Processing and Decision Making (IIPDM), University of Haifa, Haifa, Israel; 3 School of Psychological Sciences, University of Haifa, Haifa, Israel

**Keywords:** Music cognition, Sound recognition, Long-term episodic memory, Reinforcement learning

## Abstract

Music elicits powerful engagement through the generation and resolution of expectations, yet its effects on declarative memory formation remain unclear. Using predictive processing frameworks, we examined whether harmonic expectancy and prediction error modulate memory formation for concurrently presented visual stimuli. Participants listened to Bach chorales in which musical instruments probabilistically predicted consonant or dissonant endings, generating distinct expectations of outcomes. Neutral images were presented both before and after the critical musical period, and memory was tested 24 h later. At the period where the music either changed to dissonant or remained consonant, participants were prompted to rate whether the music was pleasant or unpleasant at that time. Participants showed robust sensitivity to harmonic structure, rating consonant endings as pleasant and responding faster to dissonant endings. Critically, unsigned prediction error magnitude positively predicted subsequent recognition accuracy and confidence. Whereas the relationship between prediction error and recognition accuracy did not reliably differ across temporal periods, its relationship with confidence was significantly stronger for images encoded in the anticipatory period preceding the musical transition. These findings indicate that musical expectations modulate declarative memory, with temporal specificity evident for subjective confidence, suggesting that prediction-related processes gate episodic encoding in a graded, time-dependent manner.

**Supplementary Information:**

The online version contains supplementary material available at https://doi.org/10.3758/s13423-026-03020-4.

## Introduction

Music is a universal phenomenon that exerts strong emotional responses, and plays a central role across all human societies (Belfi, 2019). Despite its abstract nature, music typically contains structured patterns that unfold over time to generate the experience of expectations and resolutions (Cheung et al., 2024; Lehne & Koelsch, 2015; Zatorre & Salimpoor, 2013). According to predictive models of music cognition, listeners implicitly learn statistical regularities in tonal and rhythmic structure, allowing them to anticipate upcoming musical events (Gebauer et al., 2012; Pearce, 2018). When these expectations are fulfilled or violated, prediction errors arise, shaping listeners’ affective and cognitive responses to the music (Cardona et al., 2020; Cheung et al., 2019). Thus, musical perception is inherently predictive, and deviations from expected outcomes are central to its emotional properties (Salimpoor et al., 2015).

Learning new information is fundamentally rooted in the principle of predictive processing, where the brain constantly engages in generating predictions and updating them according to regularities in one’s surroundings (Friston, 2010). When bottom-up input differs from predicted scenarios, a prediction error signal is generated, serving as a powerful driving force for learning (Clark, 2018). This notion is well established in non-declarative learning systems, and particularly those that involve reinforcement learning (Schultz, 2016), whereby midbrain dopaminergic neurons signal the discrepancy between expected and actual outcomes during associative learning (Schultz, 1997).

A growing body of studies in fact indicates that reward prediction errors governed by midbrain signals impact the formation and updating of declarative memories as well (Ergo et al., 2020; Pine et al., 2018). According to predictive processing and complementary learning frameworks, discrepancies between expected and actual outcomes signal the need to update internal models of the environment and can therefore prioritize information for encoding (Greve et al., 2017; Jang et al., 2019). Consistent with this view, unexpected events are often remembered better than expected events, and memory enhancements have been observed across a wide range of paradigms involving reward learning, statistical learning, associative inference, and schema violation. These findings have led to the proposal that prediction error serves as a general mechanism that regulates when new experiences are stored in memory, thereby linking learning and episodic encoding through a common computational principle.

A fundamental feature of musical structure relates to the distinction between consonance and dissonance. Consonant chords or harmonies refer to combinations of tones that are perceived as stable and pleasant, whereas dissonant intervals are combinations of tones that typically create a sense of tension instability or unpleasantness (Di Stefano et al., 2022; Lahdelma & Eerola, 2016; McDermott et al., 2010). Although cultural and experiential factors can influence the perceptive experience, listeners across a broad range of populations and musical expertise reliably discriminate between consonant and dissonant harmonies (Masataka, 2006; Popescu et al., 2019). Importantly, the physical features of consonance and dissonance engage neural systems associated not only with perception per se, but also with reward, salience, and learning (Bravo et al., 2020; Salimpoor et al., 2015).

Recent theoretical accounts suggest that musical pleasure and surprise emerge from predictive coding mechanisms, whereby the brain generates expectations about upcoming events and updates internal models when deviations occur (Koelsch et al., 2019; Quiroga-Martinez et al., 2020). Unexpected harmonic events, such as a dissonant ending following a consonance-predictive context, elicit prediction errors that recruit attentional and affective systems (Gold et al., 2019). Previous studies have demonstrated that such musical prediction errors can engage reinforcement learning mechanisms and influence decision-making behavior (Mas-Herrero & Marco-Pallares, 2025). These findings place music as a powerful tool for examining how prediction and affect interact in shaping cognitive processes.

Beyond their effects on perception and emotion, musical structures may also modulate memory encoding (Kurzom & Mendelsohn, 2022; Kurzom et al., 2025). Prior research has shown that temporal alignment with musical rhythm can enhance memory for visual and auditory stimuli (Bolger et al., 2013; Kubit & Janata, 2022). In addition, expectancy within melodic structure influences recognition memory for musical sequences (Schmuckler, 1997). Extending this work, recent studies suggest that harmonic structure and prediction violations also shape declarative memory (Kurzom & Mendelsohn, 2022). Moreover, exposure to specific chord types during encoding of word-image associations was shown to modulate subsequent memory performance (Kurzom et al., 2023), and delayed harmonic resolution, induced by prolonging musical tension before its release, has been associated with enhanced memory for concurrently presented information (Kurzom & Mendelsohn, 2022). These findings suggest that musical structure can extend beyond auditory processing and influence memory formation in cross-modal contexts.

Although previous studies have shown that musical structure and harmonic tension can influence memory formation, it remains unclear whether declarative memory is modulated by predictive computations themselves, rather than by the perceptual or affective properties of music. In particular, prior work has not dissociated learned expectancy from harmonic outcome, nor examined whether trial-by-trial prediction-error signals in a non-reward musical context influence memory for concurrently encoded information. Moreover, it is unknown whether such effects emerge during expectancy buildup or following expectancy resolution. The present study addresses these questions by independently manipulating predictive context and harmonic outcome while assessing memory for visual stimuli presented before and after musical resolution in a trial-wise manner.

The distinction between predicted and unpredicted harmonic outcomes offers a unique framework for examining the interaction between expectancy, valence, and memory. Consonant endings of musical phrases are typically rated as more pleasant than dissonant endings, yet unexpected dissonance may generate stronger prediction errors and heightened salience (Di Stefano & Spence, 2022; McDermott et al., 2010). From a predictive processing perspective, memory encoding may be modulated either by affective valence (e.g., unpleasant dissonance enhancing encoding) or by the magnitude of prediction error (e.g., unexpected harmonic deviations enhancing encoding through increased attentional allocation). Disentangling these possibilities requires independently manipulating predictive direction and harmonic outcome. Importantly, expectancy violations are not merely categorical events but vary continuously as listeners acquire and update expectations throughout the task (Pearce, 2018). Consequently, condition-level comparisons alone may not fully capture the cognitive processes underlying memory formation. Examining trial-by-trial prediction error provides a more direct measure of the degree to which a musical outcome deviates from current expectations and therefore offers a theoretically meaningful predictor of memory encoding. By combining experimental manipulations of expectancy with computational estimates of trial-wise prediction error, the present study tests whether memory formation is better explained by the valence of musical outcomes or by the magnitude of expectancy violation they evoke.

The present study investigated how harmonic prediction, gradually acquired during listening to multiple instrument-musical outcome pairings, shapes measurements of declarative memory and metamemory through mechanisms of expectancy resolution and prediction error. Distinct musical instruments served as probabilistic cues that gradually biased listeners toward anticipating either consonant or dissonant musical outcomes. This design enabled dissociation between predictive context and actual outcome, allowing us to examine whether memory encoding is modulated by the direction of expectation and the magnitude of deviation from it. Unique visual images were presented both before and after the critical musical event, enabling us to assess whether memory effects are temporally specific to the moment of expectancy resolution. Recognition memory was tested 24 h later using signal detection measures and subjective confidence ratings.

Building on predictive processing frameworks, we hypothesized that memory enhancement would be selectively expressed at the point of expectancy resolution and would scale with prediction error magnitude, which dynamically changed throughout the task. Specifically, we expected that deviations from acquired musical expectations would increase the salience of concurrently encoded stimuli, leading to enhanced memory formation of presented stimuli. Our primary theoretical prediction concerned the continuous magnitude of expectancy violation on each trial. We therefore complemented condition-level analyses with trial-wise computational estimates of prediction error.

## Methods

### Participants

Sixty-one healthy adults (15 males, 46 females), mean age = 23.67 ± 3.45 years, participated in the study. All participants reported normal hearing and normal or corrected-to-normal vision. Informed consent was obtained in accordance with the institutional review board of the School of Psychology (ID 092/24), and participants received either course credit or payment for their participation. Twenty-three participants reported some form of musical background, and nine of them indicated that they currently play a musical instrument. One participant was excluded due to missing responses on 22 of the 40 trials in the pleasantness judgment task during the encoding stage.

An a priori power analysis was conducted using G*Power for repeated-measures ANOVA (within-subject interaction). Assuming a medium effect size (f =.25), α =.05, and desired power of.80, the required sample size was estimated at *N* = 44. To account for potential exclusions due to compliance, as well as technical issues, data collection continued until the end of the academic semester and participant recruitment window, resulting in a final sample of *N* = 61 (60 after exclusion). No interim analyses or sequential stopping procedures were used to determine the final sample size.

### Auditory and visual stimuli

Participants listened to musical phrases, chosen from stimuli generated and published by Gold et al. (2019). The phrases were Bach chorales, containing Western music structures. Each phrase consisted of eight bars, lasting 25.60 s at 75 beats per minute (bpm). Five core phrases were used, each presented in two versions (consonant or dissonant ending) and in one of two timbres (mandolin or harp), yielding a total of 20 unique phrases. The dissonant endings had each note alternatingly transposed half a step higher or lower, with the soprano and tenor parts moving up first and the alto and bass parts moving down first; the consonant and dissonant versions of the stimuli were otherwise identical.

Each participant was assigned to the following conditions: For one instrument (either mandolin or harp), musical phrases ended consonantly on 75% of trials and dissonantly on 25% of trials (predicted consonance condition). For the other instrument, phrases ended dissonantly on 75% of trials and consonantly on 25% of trials (predicted dissonance condition). Thus, prediction direction (predicts consonance vs. predicts dissonance) was manipulated independently of the actual musical outcome (consonant vs. dissonant ending), allowing congruent and incongruent trials to be defined based on whether the ending matched the instrument’s predictive endings (see Fig. 1).

Each participant heard 40 phrases in total: 20 associated with instrument A and 20 with instrument B. For the instrument predicting consonant endings, 15 phrases ended consonantly and five dissonantly. For the instrument predicting dissonant endings, 15 phrases ended dissonantly and five consonantly. Overall, participants were exposed to an equal number of 20 consonant and 20 dissonant endings such that over all 30 trials were predicted and ten were unpredicted.

During each musical phrase, participants viewed a sequence of four neutral images. Images were presented at 15, 17, 21, and 23 s following phrase onset. The first two images appeared before the potential transition in musical style, whereas the latter two appeared after the transition, enabling a temporal manipulation relative to the critical musical event (before vs. after change). The images were drawn from the neutral subset of the International Affective Picture system (IAPS) database. Each image was presented for 1 s during encoding and appeared only once during the experiment.

### Task procedure

At the beginning of the encoding session, participants were informed that they would hear short musical phrases while viewing images on the screen and that their memory for the images would be tested the following day. They were instructed to maintain fixation on a central fixation cross throughout each trial while performing a pleasantness judgment task. Nineteen seconds after the onset of each musical phrase, the prompt “Pleasant? Yes/No” appeared on the screen. Participants indicated whether they thought the musical phrase to be pleasant or unpleasant by pressing the corresponding key on the keyboard. The response categories were explained before the task: a “Yes” response indicated that the phrase remained harmonically coherent and continuous, whereas a “No” response indicated that the phrase ended in an off-key, altered, or harmonically incongruent manner. To familiarize participants with the distinction between consonant and dissonant endings, they first completed a brief practice session consisting of two trials: one with a consonant ending and one with a dissonant ending.

### Recognition memory test

Approximately 24 h after encoding, participants completed a recognition memory test. The test consisted of all previously presented images (160 old items) and an equal number of novel images (new items). Images were selected at random for each participant and each condition. For each image, participants made an old/new judgment (“familiar” vs. “unfamiliar”) and provided a confidence rating regarding their judgment on a continuous scale ranging from “Uncertain,” “Somewhat certain,” to “Very Certain.”

### Behavioral data analysis

**Fig. 1:**
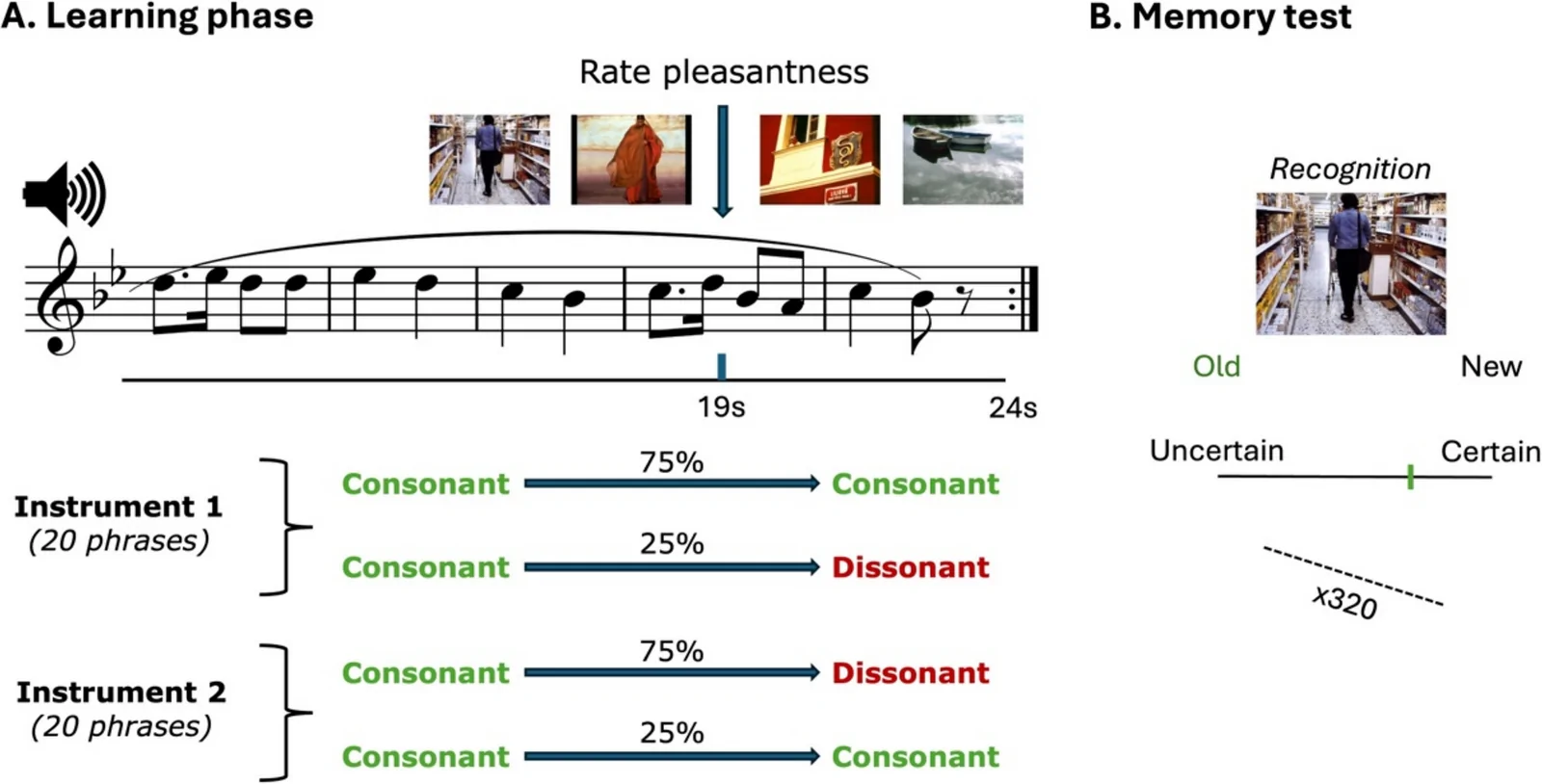
Experimental design and probabilistic manipulation of musical expectations. **A** Participants listened to 24-s musical phrases while viewing neutral images presented sequentially during encoding. Two images appeared before the critical musical ending (19 s from phrase onset), and two appeared after the ending (final 5 s). At the time of the musical change, participants were visually prompted to rate the pleasantness of the phrase by pressing one of two buttons (pleasant/unpleasant). Two instruments served as predictive cues. For one of the instruments, phrases ended consonantly on 75% of trials and dissonantly on 25% of trials (predicted consonance condition). For the other instrument, phrases ended dissonantly on 75% of trials and consonantly on 25% of trials (predicted dissonance condition). This manipulation created congruent trials (endings that matched the instrument’s predictive direction) and incongruent trials (endings that violated the predictive bias), allowing prediction direction (consonance vs. dissonance) and congruency (congruent vs. incongruent) to be examined independently. Each instrument was presented in 20 phrases. **B** Approximately 24 h later, participants returned for a recognition memory test. All 160 images presented during the encoding phase, along with 160 novel images, were shown sequentially in randomized order. For each image, participants indicated whether it was previously seen (“old”) or not (“new”), and rated their confidence in that decision

### Reaction time analysis

Reaction times (RTs) from the pleasantness judgment task were analyzed to examine sensitivity to musical ending type, predictive congruency, and learning across the experiment. For each participant, median RTs were calculated for each of the four conditions defined by Prediction Direction and Musical Outcome: predicted consonant–consonant outcome (congruent), predicted consonant–dissonant outcome (incongruent), predicted dissonant–dissonant outcome (congruent), and predicted dissonant–consonant outcome (incongruent). Congruent trials were binned to include three trials in each, yielding a similar number of congruent (bins) and incongruent trials.

Trial-level data were entered into a linear mixed-effects model using MATLAB’s *fitlme* function. Fixed effects included Bin (modeled continuously), Ending (consonant, dissonant), Congruency (congruent, incongruent), and all interactions. Subject was included as a random effect with random intercepts and random slopes for Bin: RT ~ Bin * Ending * Congruency + (1 + Bin | Subject). ANOVA tests of fixed effects were computed using residual degrees of freedom (DFMethod = Residual).

### Recognition memory analysis (signal detection measures)

Recognition performance was quantified using d′ (d-prime), computed separately for each participant and condition. Hit rates and false alarm rates were calculated, and loglinear corrections were applied where necessary to avoid infinite values. Primary analyses focused on three within-subject factors: Temporal position of the image relative to the musical change (before vs. after), prediction direction (predicts consonance vs. predicts dissonance), and congruency (congruent vs. incongruent, defined as whether the musical ending matched the instrument’s predictive bias).

For both d' and normalized confidence ratings, a 2 × 2 × 2 repeated-measures ANOVA was used with the within-subject factors of Time (before vs. after the musical change), Prediction Direction (predicts consonance vs. predicts dissonance), and Congruency (congruent vs. incongruent). Given the d' findings in this omnibus analysis, follow-up analyses were conducted only on images presented after the musical change. A 2 × 2 repeated-measures ANOVA was performed on post-change d′ values with the within-subject factors of Prediction direction and Congruency. This analysis tested whether post-event memory sensitivity differed as a function of predictive context and congruency.

### Trial-by-trial prediction error analysis

Prediction error (PE) values were computed on a trial-by-trial basis using a simple Rescorla–Wagner reinforcement learning model (Fig. S1, Online Supplementary Material). Expected probabilities for each musical ending type were updated separately for each instrument cue according to:

$${V}_{t+1}={V}_{t}+\alpha *{\delta}_{t},$$

where V_t_ represents the expected outcome value of a given ending on trial t, α is the learning rate, and δ is the prediction error, computed as the difference between outcome and expected outcome value: δ_t_ = O_t_ – V_t_.

Values were initiated at zero, and O (outcome) was coded as 0.5 for consonant endings and −0.5 for dissonant endings. Unsigned prediction error was defined as the absolute value of the prediction error term, reflecting the magnitude of expectancy violation irrespective of outcome valence. A fixed learning rate (α = 0.2) was used for all participants rather than estimating subject-specific parameters, as the goal of the model was to derive trial-wise estimates of expectancy violation from the experimental contingencies rather than to optimize individual behavioral fits.

Separate outcome values were calculated for each instrument cue, reflecting the learned association between that instrument and the likelihood of consonant versus dissonant endings. On each trial, prediction error was computed using the expectation associated with the instrument presented on that trial and the actual musical outcome that followed. The resulting trial-wise unsigned prediction error value was assigned to all images presented during that musical phrase and entered as a predictor in the trial-level memory analyses.

To visualize and characterize the relationship between prediction error and memory performance (separately for hit-rates and confidence), a binning procedure was applied to trial-level data. Because incongruent trials were relatively sparse and did not provide enough trials for stable bin-level results, binning analyses were conducted using only congruent trials.

Unsigned prediction error values were pooled across participants and sorted into three equally spaced bins spanning the observed prediction error range. For each bin, memory performance (hit rate) and confidence were computed separately for each participant. Group-level means were then calculated by averaging across participants within each bin. Shaded error bands represent ± 1 SEM across participants. This binning procedure was used for visualization and descriptive purposes only; all statistical inferences were conducted using trial-level mixed-effects models.

### Trial-level mixed-effects modeling

To examine whether trial-by-trial musical prediction error influenced subsequent memory performance and confidence ratings, we conducted mixed-effects analyses in MATLAB using the fitglme and fitlme functions, respectively. Prediction errors were standardized within each participant (zPE) prior to analysis. Two dependent variables were analyzed: Recognition accuracy (binary outcome: hit = 1, miss = 0), which was analyzed using logistic mixed-effects regression, and Recognition confidence (continuous rating scale), analyzed using linear mixed-effects models.

For each outcome, we first estimated a model testing the effect of prediction error:

$$Accuracy/Confidence \sim 1 + zPE + \left(1 + zPE\left|Subject\right.\right)$$

We then examined whether the relationship between prediction error and memory differed as a function of the temporal position of the encoded image relative to the musical ending (Before vs. After) by adding Time and its interaction with prediction error:

$$Accuracy/Confidence \sim 1 + zPE*Time+\left(1+zPE\left|Subject\right.\right)$$

Random intercepts and random slopes for prediction error were included for each participant in all models. The critical effect of interest was the Prediction Error × Time interaction, which tested whether the influence of prediction error on memory differed for images presented before versus after the musical ending.

## Results

Analyses were conducted in three steps. First, reaction time (RT) data were examined to assess online sensitivity to musical changes, revealing faster detection of dissonant endings. Second, recognition memory was analyzed using signal detection measures, followed by item-level mixed-effects models, which were used to assess the robustness of these effects across subjects and items.

### Pleasantness ratings and reaction times (RTs)

**Fig. 2:**
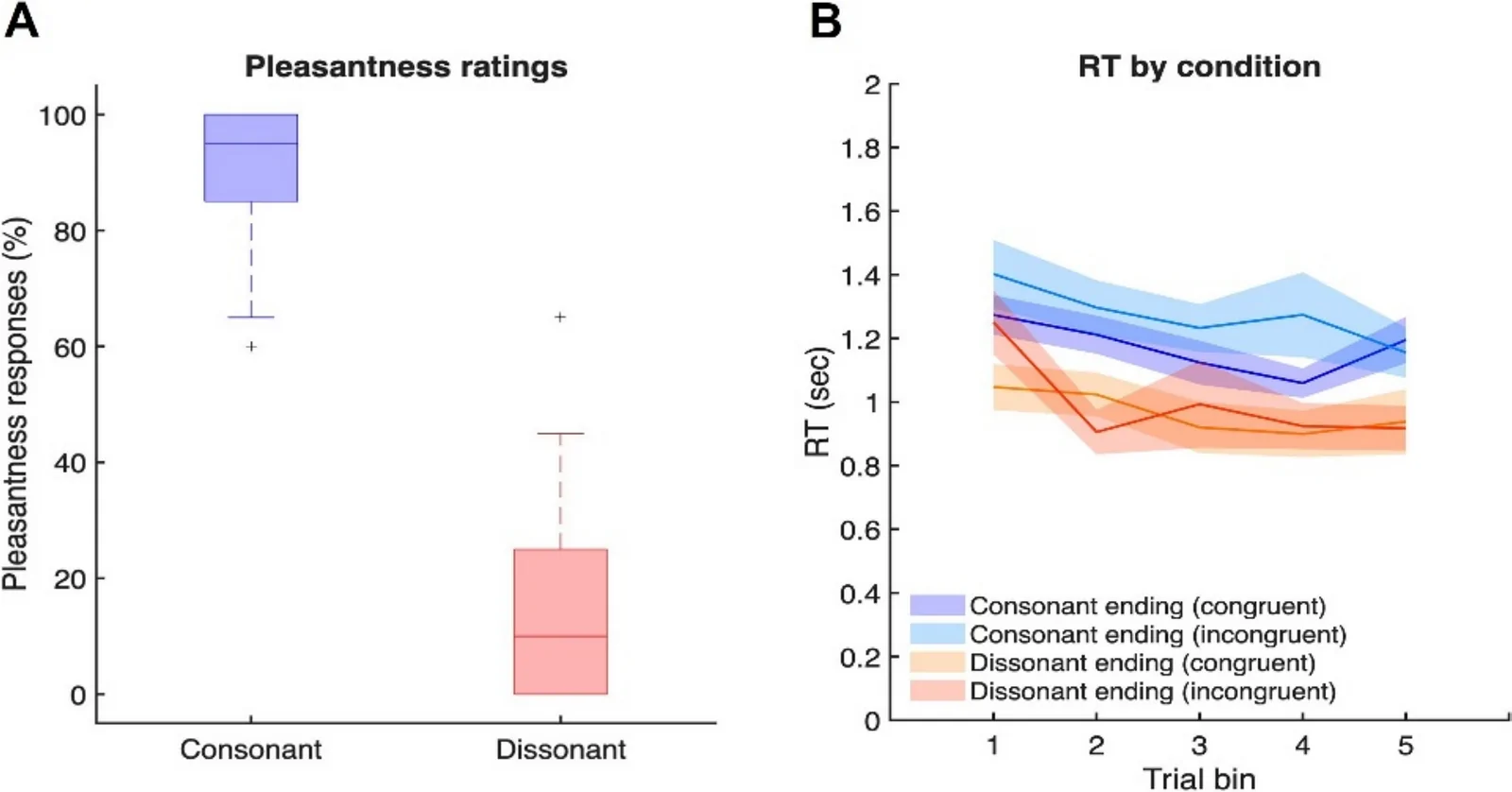
Behavioral responses during the learning phase.** A** Pleasantness ratings as a function of musical ending type. Consonant endings were rated as pleasant on a high proportion of trials, whereas dissonant endings were predominantly rated as unpleasant. Boxplots depict the median, interquartile range, and range across participants.** B** Reaction times (RTs) for pleasantness judgments as a function of trial bin and condition. RTs are shown separately for consonant and dissonant endings and for congruent versus incongruent trials. Responses were faster for dissonant compared to consonant endings across bins. Shaded areas represent ± 1 *SEM* across participants

For each phrase, participants listened to the music, and after 19 s from beginning were prompted to indicate whether the music was pleasant or not. As shown in Fig. 2A, accuracy levels were overwhelming; a mean of 84.416% ± 2.456 of consonant endings were rated as pleasant, as opposed to 17.083% ± 2.697 for dissonant endings (*t*(59) = 15.814, *P* < 0.0001).

RT analysis revealed that there was no significant main effect of Bin, *F*(1, 1166) = 2.82, *p* =.093, *ηp*^*2*^ =.002, indicating no reliable change in RT across the experiment (Fig. 2B). A significant main effect of Ending was observed, *F*(1, 1166) = 5.50, *p* =.019, *ηp*^*2*^ =.005, such that responses differed between consonant and dissonant endings. Participants responded faster to dissonant relative to consonant endings. The main effect of Congruency was not significant, *F*(1, 1166) = 0.51, *p* =.474, *ηp*^*2*^ <.001. Importantly, a significant Ending × Congruency interaction was found, *F*(1, 1166) = 10.52, *p* =.0012, *ηp*^*2*^ =.009, indicating that the effect of congruency differed depending on the musical ending type.

### Condition-level memory and confidence

#### Recognition memory (d’)

**Fig. 3:**
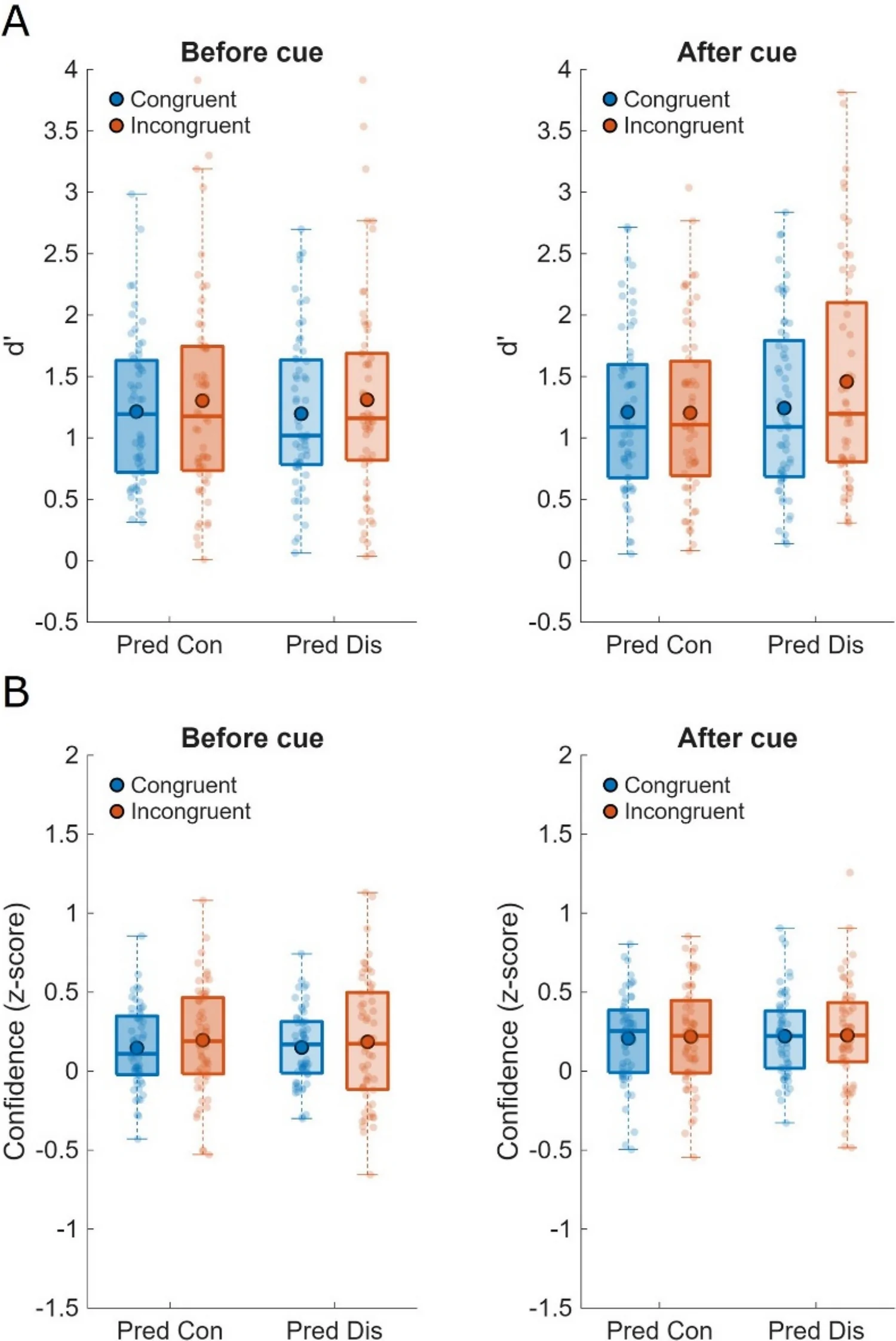
Effects of prediction direction and congruency on recognition memory and confidence before and after the rating cue. **A** Recognition sensitivity (d′) as a function of prediction direction (Pred Con = instrument predicting consonance; Pred Dis = instrument predicting dissonance), congruency (Congruent vs. Incongruent), and temporal position relative to the musical ending (Before change vs. After change). **B** Recognition confidence (z-scored) plotted using the same factors. Boxplots display the interquartile range (IQR) with the median indicated by the horizontal line. Whiskers extend to 1.5 × IQR. Dots represent individual participants. Colored markers denote condition means. Congruency is defined relative to the instrument’s predictive bias (i.e., whether the musical ending matched the learned probability associated with the instrument)

A 2 × 2 × 2 repeated-measures ANOVA was conducted on d′ values (Fig. 3A) with the within-subject factors of Time (before vs. after the musical change), Prediction Direction (predicts consonance vs. predicts dissonance), and Congruency (ended as expected by musical instrument vs. ended differently than expected). There was no main effect of Time, *F*(1, 59) = 0.31, *p* =.583, *ηp*^*2*^ =.005, indicating that on average, memory sensitivity did not differ between images presented before versus after the musical change. The main effect of Prediction Direction was not significant either, *F*(1, 59) = 1.42, *p* =.238, *ηp*^*2*^ =.023, suggesting that memory sensitivity did not differ reliably between contexts predicting consonance versus dissonance.

The main effect of Congruency approached significance, *F*(1, 59) = 3.87, *p* =.054, *ηp*^*2*^ =.062, indicating a trend toward differential memory performance for congruent compared to incongruent trials. A significant Time × Prediction Direction interaction was observed (*F*(1, 59) = 4.54, *p* =.037, *ηp*^*2*^ =.071), demonstrating that beyond congruency, the influence of predictive context on memory depended on whether images were presented before or after the musical change. No other interactions were significant.

To further explore the significant Time × Prediction Direction interaction observed in the omnibus analysis, separate 2 × 2 repeated-measures ANOVAs (Congruency × Prediction Direction) were conducted for pre- and post-change trials. For images presented before the musical change, neither the main effects nor the interaction reached significance (all *p*s >.10), indicating no reliable modulation of memory prior to the critical musical event. In contrast, for images presented after the change, a significant main effect of Prediction Direction was found, *F*(1, 42) = 5.36, *p* =.026, *ηp*^*2*^ =.113, while other effects were not significant.

Taken together, these results indicate that, at the level of condition-averaged d′ scores, recognition memory was not uniformly modulated by congruency or prediction direction and was influenced primarily by the temporal relationship between image presentation and the musical event. However, because these analyses rely on averages computed across trials within each condition, they do not capture variability in the magnitude of prediction error or differences in subjective valence across individual events. To examine whether memory was sensitive to these graded, trial-level properties of the musical experience, we conducted analyses incorporating computational estimates of prediction error and value.

#### Confidence ratings

Subjective recognition experience computed from confidence-based responses was analyzed using a 2 × 2 × 2 repeated-measures ANOVA with the within-subject factors Temporal Position (Before vs. After), Prediction Direction (predicts consonance vs. predicts dissonance), and Congruency (congruent vs. incongruent) (Fig. 3B). No significant main effects were observed for Temporal Position, *F*(1,58) = 2.87, *p* =.096, Prediction Direction, *F*(1,58) = 0.01, *p* =.914, or Congruency, *F*(1,58) = 1.00, *p* =.322. Thus, confidence-based memory sensitivity did not differ reliably as a function of time relative to the musical change, predictive context, or congruency.

### Prediction error effects on recognition accuracy

To examine whether musical prediction error influenced subsequent memory, we first fitted a linear mixed-effects model predicting trial-level recognition accuracy from z-scored unsigned prediction error (z absolute PE), with random intercepts and slopes for prediction error across participants (see Fig. S1 in the Online Supplementary Material for trial-by-trial prediction error examples and absolute PE distribution across the sample). This analysis revealed a significant positive effect of prediction error on recognition accuracy, *b* = 0.058, *SE* = 0.027, *t*(7198) = 2.15, *p* =.032, 95% CI [0.005, 0.111]. Thus, larger deviations from learned musical expectations were associated with an increased likelihood of correctly recognizing the associated visual stimulus at test. Random-effects estimates indicated substantial between-subject variability in overall recognition performance, as well as smaller but reliable variability in sensitivity to prediction error.

**Fig. 4:**
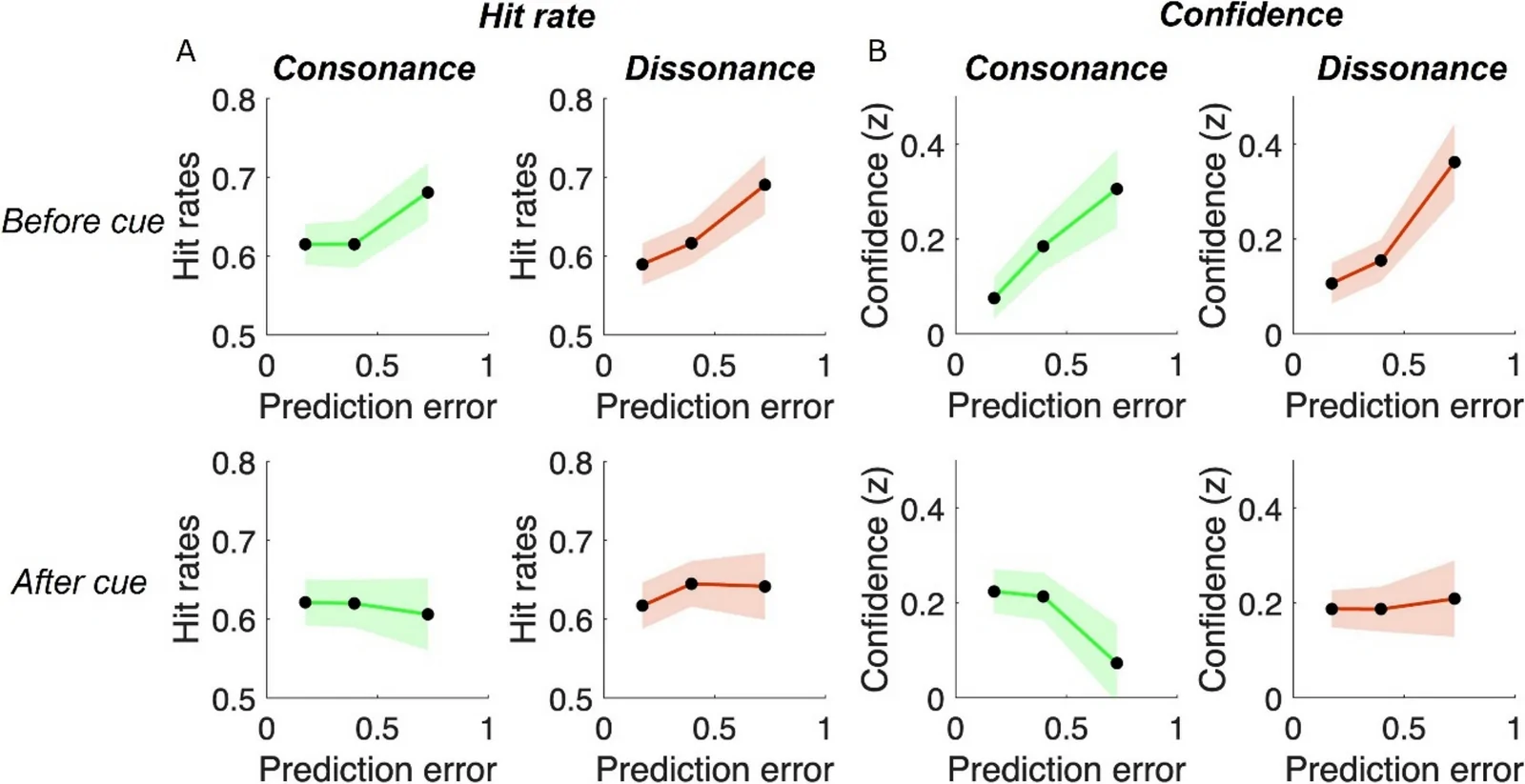
Effects of prediction error on memory performance and confidence before and after contingency change. **A** Hit rates as a function of prediction error for consonant (green) and dissonant (orange) trials. Before the rating cue (top row), hit rates increased with prediction error for both trial types. After the rating cue (bottom row), this relationship was attenuated for consonant trials and remained relatively stable or slightly elevated for dissonant trials. **B** Confidence ratings (z-scored) plotted against prediction error for consonant (green) and dissonant (orange) trials. Before the change, confidence increased with prediction error in both conditions. After the change, confidence decreased with prediction error for consonant trials, whereas dissonant trials showed little change across prediction error levels. Points represent mean values; shaded regions indicate ± *SEM*

To determine whether this effect depended on the temporal position of the encoded image relative to the musical ending, we extended the model to include Time (Before vs. After the change) and its interaction with prediction error. In this model, prediction error remained a positive predictor of recognition accuracy (*b* = 0.099, *SE* = 0.038, *t*(7196) = 2.64, *p* =.008). However, the interaction between prediction error and Time was not significant (*b* =  − 0.082, *SE* = 0.052, *t*(7196) =  − 1.58, *p* =.113). Model comparison indicated no improvement in fit with the inclusion of Time (ΔAIC = 8), suggesting that the influence of prediction error on recognition accuracy did not differ reliably before versus after the musical event. Together, these findings indicate that prediction error enhances memory accuracy at the trial level, and that this effect is observed for images encoded both before and after the musical change (Fig. 4).

### Prediction error effects on confidence

We next examined whether prediction error influenced subjective memory confidence. A mixed-effects model predicting confidence ratings from prediction error revealed a significant positive effect, *b* = 0.014, *SE* = 0.0069, *t*(7187) = 2.08, *p* =.038, 95% CI [0.0008, 0.0281], demonstrating that higher prediction errors were associated with increased subjective confidence in recognition answers.

Crucially, when Time and its interaction with prediction error were included in the model, a significant interaction emerged. In the full model, prediction error remained a robust positive predictor of confidence (*b* = 0.032, *SE* = 0.0093, *t*(7185) = 3.48, *p* =.0005), and there was also a main effect of Time (*b* = 0.032, *SE* = 0.0124, *t*(7185) = 2.54, *p* =.011), indicating overall higher confidence for images presented after the musical change. Importantly, the interaction between prediction error and Time was significant (*b* =  − 0.036, *SE* = 0.0124, *t*(7185) =  − 2.89, *p* =.0039). Model fit improved with the inclusion of Time and its interaction (ΔAIC =  − 10), supporting the presence of a temporally specific modulation of the prediction error effect on confidence. The negative coefficient indicates that the positive relationship between prediction error and confidence was significantly stronger for images presented before the musical ending and attenuated for images presented after the ending. In other words, prediction error was more robustly associated with increased confidence during the pre-change period, whereas this relationship was reduced following the musical resolution.

Similar analysis both for hit rates and confidence did not yield any significant findings when testing value instead of prediction error.

## Discussion

The present study examined whether musical expectancy and prediction error modulate declarative memory formation for concurrently presented visual stimuli. We assessed the influences of predictive probability (instrument-based contingencies) and harmonic outcome (consonant vs. dissonant endings) on memory formation of visual stimuli that were presented at varying time-points relative to expectancy resolution. This design enabled us to test whether long-term memory is primarily driven by expected outcome value, congruency, or prediction error-related signals. The results showed that trial-by-trial prediction error was associated with both subsequent recognition accuracy and confidence, while temporal specificity was evident primarily for confidence, for which the prediction error relationship was stronger for information presented before rather than after musical resolution.

### Online sensitivity to harmonic deviations

Behaviorally, participants demonstrated robust sensitivity to harmonic features of the musical phrases, consistently rating consonant endings as pleasant and dissonant as unpleasant. This replicates well-established findings regarding the affective distinction between consonance and dissonance (Koelsch, 2014; Lahdelma & Eerola, 2016). RTs further revealed faster responses to dissonant compared to consonant endings, suggesting heightened salience or attentional capture by dissonant outcomes, regardless of musical expectations. These findings confirm that the musical manipulation effectively engaged perceptual and evaluative systems and provided the basis for testing how musical expectancies related to memory performance.

Previous research has demonstrated that prediction errors arising from musical expectations reflect the interaction between expectancy and affective value (Gold et al., 2019). In Gold et al. (2019), prediction errors were positive for consonant outcomes and negative for dissonant outcomes, with error magnitude increasing as outcomes deviated more strongly from learned expectations. Consonance and dissonance have therefore been operationalized as musical analogues of reinforcement outcomes within computational reinforcement learning frameworks (Mas-Herrero & Marco-Pallares, 2025; Quiroga-Martinez et al., 2019).

Using computational modeling and neuroimaging, Gold et al. (2019) demonstrated that musical outcomes elicited reinforcement prediction errors (RPEs) that tracked both expectancy and pleasantness. Importantly, RPE-related activity in reward-related regions, particularly the nucleus accumbens, predicted musical learning performance, such that individuals with stronger activation showed greater predictive accuracy over time. These findings demonstrate that music, even in the absence of explicit rewards, can generate physiological reinforcement signals that can guide learning and decision-making. Consistent with these findings, our results indicate that musically induced expectations also modulate declarative memory formation. Although our paradigm did not involve explicit reward-based decision making, the probabilistic associations between instrument and ending generated learned expectations that influenced subsequent recognition performance. Specifically, using the musical phrases originated by Gold et al. (2019), we found that memory performance, as indicated by both correct recognition and metamemory judgments, corresponded with unsigned PE, even before the musical change. These effects suggest that memory encoding is modulated by expectancy of musical events, in line with the idea that music can generate reinforcement-like prediction signals that guide learning even in the absence of explicit reward (Koelsch et al., 2019).

### Temporal specificity of memory modulation

The present findings indicate that musical prediction errors are associated with heightened memory and subjective confidence ratings. Importantly, and particularly for subjective memory confidence, this effect was expressed more strongly for images encoded before the critical epoch of musical ending. Larger unsigned deviations from learned harmonic expectations were associated with better subsequent recognition and confidence, consistent with reinforcement-learning and predictive-coding accounts in which prediction errors act as teaching signals that prioritize concurrent information for encoding (Lisman & Grace, 2005; Yifrah et al., 2025). Prior work has shown that reward-related and contextual prediction errors can enhance declarative memory (Pine et al., 2018; Rouhani et al., 2018). Our results extend these principles to the musical domain and suggest that anticipatory violations of harmonic expectation influence memory and metamemory even in the absence of explicit reward.

Importantly, the PE–memory relationship was attenuated for images presented after the musical change, indicating that the mnemonic benefit was strongest in the anticipatory window preceding outcome resolution. This pattern is consistent with theoretical accounts emphasizing the role of uncertainty and expectation buildup in modulating attention and encoding (Huron, 2006; Tillmann et al., 2014). Notably, because memory performance scaled with the magnitude of the prediction error ultimately generated on each trial, the observed effects cannot be fully explained by expectancy or anticipatory attention. Rather, the findings suggest that PE-related memory enhancements may emerge from processes engaged during prediction formation and maintenance, before outcome resolution occurs, rather than exclusively from post-outcome model updating.

This notion resonates with pre-PE memory enhancement effects reported outside the realm of music. A recent study showed that prediction-error-related memory enhancements depend not only on the outcome itself but also on neural states immediately preceding the prediction error event (Loock et al., 2025a, 2025b). Similarly, expectations regarding the category of upcoming visual stimuli have been shown to enhance subsequent recognition memory for individual items. Together, these findings suggest that preparatory expectancy-related processes contribute to the relationship between prediction error and memory. In the present study, memory benefits may scale with the uncertainty of the predicted outcome, which in turn determines the magnitude of the prediction error once the outcome is revealed.

A complementary interpretation concerns cross-modal attentional allocation. Because prediction errors were induced in the auditory domain whereas memory was tested for visual stimuli, expectancy violations may have redirected attentional resources toward processing the musical stream itself (Kurzom & Mendelsohn, 2022). More generally, attentional competition between auditory and visual inputs is a well-established feature of multisensory processing and is not specific to music (Fernandes & Moscovitch, 2000; Johnson & Zatorre, 2006). Under this account, anticipatory engagement during the unfolding musical phrase may have supported encoding of concurrently presented visual stimuli, whereas processing demands associated with the resolution of the musical outcome could have reduced resources available for visual encoding after the critical event. Such a mechanism would predict stronger memory effects for images encountered before, rather than following, the musical resolution.

At the same time, this attentional account does not fully explain the present findings, as memory performance was related to the magnitude of the trial-by-trial prediction error ultimately generated on each trial. Thus, while cross-modal attentional allocation may contribute to the observed temporal profile, the results suggest that attentional processes likely interact with predictive computations rather than providing a complete alternative explanation. A related possibility is that the musical resolution also functioned as an event boundary, with the transition altering the contextual representation of information encountered before versus after the musical change. Event-segmentation accounts propose that such contextual transitions can reorganize the encoding of information (Davachi & DuBrow, 2015; Zacks et al., 2007), and recent work suggests that contextual stability may sometimes account for segmentation-related memory effects more strongly than prediction error itself (Güler et al., 2025). However, since prediction error and contextual change were not independently manipulated here, future studies could distinguish their respective contributions to the observed temporal effects.

Together, these findings suggest that anticipatory predictive states may be influential in shaping both memory strength and metacognitive evaluation. Crucially, expected value did not predict accuracy or confidence, suggesting that expectancy violation, rather than stimulus value per se, drove the observed effects. Future studies incorporating neuroimaging or physiological measures could directly test whether dopaminergic or hippocampal activity mediates the observed behavioral effects. Second, the present study focused on harmonic expectancy; extending this paradigm to other dimensions of musical structure (e.g., rhythm, tempo, timbre) could clarify whether prediction-driven memory enhancement is a domain-general phenomenon within music cognition.

The present findings demonstrate that musical expectations modulate declarative memory formation, with prediction error magnitude predicting subsequent recognition accuracy and confidence. Temporal specificity was particularly evident for subjective confidence, for which the relationship with PE was strongest during the anticipatory period preceding musical resolution. These results suggest that musical expectancy can act as a gating mechanism for episodic encoding, highlighting a broader role for predictive processing in shaping memory formation across modalities.

## Supplementary Information

Below is the link to the electronic supplementary material.

Supplementary file1 (DOCX 173 kb)

## Data Availability

All data and materials have been made publicly available via the OSF and can be accessed at https://osf.io/vbmjc/overview/. Analysis scripts: Analysis scripts are available from the corresponding author upon reasonable request.

## Declarations

### Open practices statement

This study was not pre-registered.
