# Supplementary material for: Musical expectations modulate declarative memory formation
Source: Psychon Bull Rev. 2026 Sep 23;33(7):245. doi: 10.3758/s13423-026-03020-4 (PMC13601126; doi:10.3758/s13423-026-03020-4)
Supplement: Supplementary file 1 [file 13423_2026_3020_MOESM1_ESM.docx]

Supplementary Material

**Musical expectations modulate declarative memory formation**

**Figure S1. Examples of trial-by-trial prediction error estimates and distribution of unsigned prediction error values.** **(A)** Example prediction error (PE) values generated by the Rescorla–Wagner model for a representative participant, shown separately for the instrument predominantly associated with consonant endings (left) and the instrument predominantly associated with dissonant endings (right). Prediction error values were updated on each trial based on the discrepancy between the expected and observed musical outcome, illustrating the dynamic evolution of expectancy throughout the task. **(B)** Distribution of unsigned (absolute) prediction error values across all trials and participants. Dashed vertical lines indicate the 33rd and 67th percentiles, which were used to divide prediction error values into three equally sized bins for visualization of the relationship between prediction error and subsequent memory performance (shown in Figure 4 of the main text).
